# Transporting Cells in Semi-Solid Gel Condition and at Ambient Temperature

**DOI:** 10.1371/journal.pone.0128229

**Published:** 2015-06-22

**Authors:** Junjian Wang, Peng Chen, Jianzhen Xu, June.X Zou, Haibin Wang, Hong-Wu Chen

**Affiliations:** 1 Comprehensive Cancer Center, University of California Davis, Sacramento, California, 95817, United States of America; 2 Department of Biochemistry and Molecular Medicine, School of Medicine, University of California Davis, Sacramento, California, 95817, United States of America; 3 First Clinical Medicine School of Guangzhou University of Chinese Medicine, Guangzhou, 510405, China; 4 Shantou University Medical College, No. 22 Xinling Road, Shantou, 515041, China; 5 First Affiliated Hospital of Guangzhou University of Chinese Medicine, Guangzhou, 510405, China; Genomics Institute Novartis Research Foundation, UNITED STATES

## Abstract

Mammalian cells including human cancer cells are usually transported in cryovials on dry ice or in a liquid nitrogen vapor shipping vessel between different places at long distance. The hazardous nature of dry ice and liquid nitrogen, and the associated high shipping cost strongly limit their routine use. In this study, we tested the viability and properties of cells after being preserved or shipped over long distance in Matrigel mixture for different days. Our results showed that cells mixed with Matrigel at suitable ratios maintained excellent viability (>90%) for one week at room temperature and preserved the properties such as morphology, drug sensitivity and metabolism well, which was comparable to cells cryopreserved in liquid nitrogen. We also sent cells in the Matrigel mixture via FedEx service to different places at ambient temperature. Upon arrival, it was found that over 90% of the cells were viable and grew well after replating. These data collectively suggested that our Matrigel-based method was highly convenient for shipping live cells for long distances in semi-solid gel condition and at ambient temperature.

## Introduction

Mammalian cells including the large number of cell lines are widely used in scientific research and drug discovery. Transporting cells often occurs around the world. Therefore, convenient and low-cost methods are desirable for routine transportation of mammalian cells at long distance. One widely used method of transporting cells is shipping cryopreserved cells on dry ice[1,2]. However, dry ice is a hazardous material for its possible cause of explosion and suffocation. Based on Federal Express shipping information (http://www.fedex.com/us/dangerous-goods/index.html), more than 50% (114/200) of countries around the world prohibit delivery with dry ice, which limits transporting cells in different countries by this method. Moreover, dry ice in the routinely used shipping containers can only last for several days at most. Due to the high-cost, liquid nitrogen vapor shippers are usually used for high valuable biomaterials. Shipping cells in a flask filled with complete growth medium at ambient temperature is an alternative method [3], but is only suitable for transporting cells across a short distance and over a very short period of time (usually up to 24 hours) [4]. Many types of cells do not survive well through shipping in flasks with medium.

**Fig 1:**
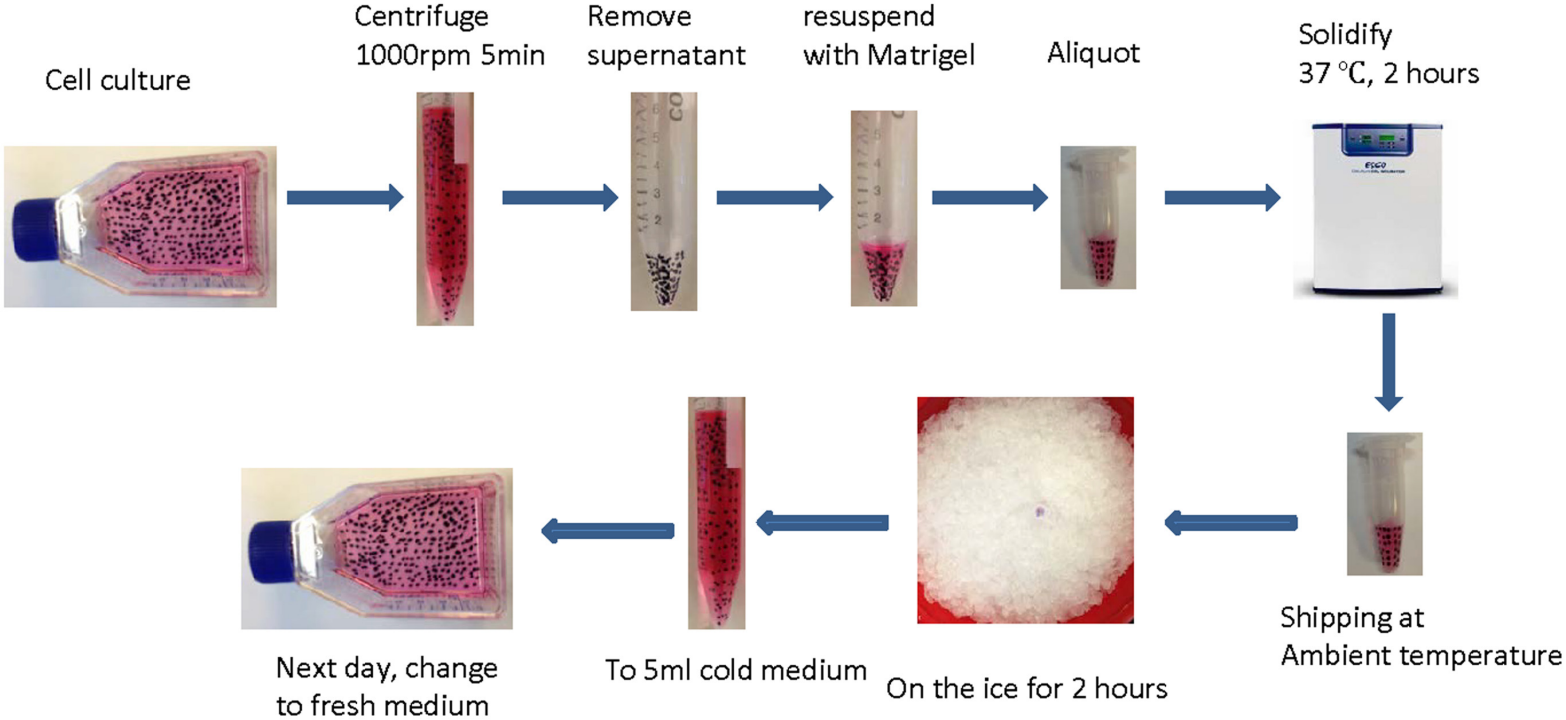
A flowchart of the Matrigel based method for transporting cells in semi-solid gel condition and at ambient temperature.

Matrigel is a gelatinous protein mixture secreted by Engelbreth-Holm-Swarm (EHS) mouse sarcoma cells. It is composed of primarily proteins such as as laminin, entactin, collagen, and proteoglycan with cell adhesive peptide sequences, and small amount of growth factors, which together promotes the survival of many type of cells [5]. Matrigel is widely used in culturing cells for their optimal growth under certain conditions, in cell invasion assays, and in establishment of xenograft tumors in immuno-deficient mice [5–8]. Matrigel can be frozen at -20°C or lower temperatures, liquefied in a 4°C refrigerator or on wet ice, and solidified at above 10°C. Given these properties of Matrigel, here we have developed a Matrigel-based method for transporting cells in semi-solid gel condition and at ambient temperature (Fig 1).

## Materials and Methods

### Cell culture and reagents

All cell lines except SUM159 (gift from Dr. John Albeck) were obtained from ATCC (Manassas, VA, USA)[9]. Human lung cancer cell line A549 and osteosarcoma cell line U2OS cells were cultured in RPMI 1640 medium (Invitrogen, Grand Island, NY, USA). MDA-MB231 and MCF-7 human breast cancer cells were cultured in DMEM medium (Invitrogen, Grand Island, NY, USA). Human breast cancer cell line SUM159 cells were cultured in F12 medium (Invitrogen, Grand Island, NY, USA) containing 5μg/L insulin and 1μg/L Hydrocortisone. Cell culture media were supplemented with 1% penicillin/streptomycin and 10% fetal bovine serum (Gemini Bio Products, West Sacramento, CA, USA). Cells were cultured at 37°C under 5% CO_2_ in a humidified incubator. In experiments involving treatment with 17β-estradiol (E2) or ethanol control, cells were placed in estrogen-deprived medium consisting of phenol red-free DMEM with 10% dextran–charcoal-stripped fetal bovine serum for 2 days prior to the addition of hormone or vehicle. Cells recovered from the medium-supplemented Matrigel or thawed from cryopreserved aliquots stored in liquid nitrogen were passaged three times before used in the assays. Matrigel Matrix (Catalog No. #356234) was purchased from BD Biosicences. All chemicals were purchased from Sigma-Aldrich (St. Louis, MO) unless specified otherwise.

### Procedures of storing and recovering cells in medium-supplemented Matrigel

For storing and shipping cells in medium-supplemented Matrigel, the following procedures were followed:

1. Let the Matrigel liquefy overnight at 4°C.
2. Mix the Matrigel with cell growth medium (cold on the ice) at desired ratios and keep them on the ice before use.
3. Detach cells from the flask or plate with trypsin-EDTA before centrifugation at low speed.
4. Remove supernatant and resuspend the cell pellet with 1.2 ml mixture of Matrigel and media.
5. Aliquot the resuspended cells to 1.5ml Eppendorf tubes (300μL per tube), and place them at 37°C under 5% CO_2_ in a humidified incubator for 2 hours to allow the Matrigel to solidify.
6. Move the tubes out of the incubator for shipping at ambient temperature.

For recovering cells:

1. Place the tubes on ice for two hours to allow the mixture to liquefy.
2. Add 0.7ml cold cell growth medium to the tube and gently mix by pipetting.
3. Transfer the content to a 15ml tube containing 9ml cold cell growth medium, followed by gentle and brief pipetting to mix.
4. Transfer the content by pipette to the flask or plate, incubate at 37°C under 5% CO_2_ in a humidified incubator for 24 hours before changing to fresh growth medium.

### Freezing and thawing cells in cryopreservation vials

Conventional procedures were followed. Briefly, cells were harvested, resuspended in freezing media (10% DMSO and 90% complete cell growth medium) at 5.0 x 10^6^ cells per ml, aliquoted into cryogenic vials (1 ml/vials). The vials were then placed in a Freezing Container (Nalgene Cryo 1°C, Thermo Scientific) overnight at -80°C before transferring vials to liquid nitrogen storage tank. For thawing/awaking the cells, frozen vials were quickly thawed in a 37°C water bath with gentle shaking. The content was mixed with 9 ml cell growth medium before centrifugation. The cell pellet was resuspended in fresh cell growth medium and transferred to a flask for culturing. The medium was changed 24 hours later.

### Trypan blue exclusion cell viability assay

Cell viability was determined using a standard trypan blue dye exclusion method (0.4% trypan blue, Life Technologies, USA) according to instructions of manufacture. Briefly, cells were recovered from medium-supplemented Matrigel or thawed from cryopreserved vials, and mixed with 10 ml complete cell growth medium. Aliquots containing 50 μl cell growth medium were gently mixed with an identical volume of 0.4% trypan blue. The number of viable cells was subsequently determined under light microscope. Cell viability was calculated as the percentage of viable cells out of the total number of cells initially preserved.

### Cell growth and colony formation assays

Cells recovered from either the Matrigel tube or cryogenic vial were cultured as described above. For cell growth, cells were trypsinized and seeded into 6-well plates at 2 × 10^5^ per well when they grow to ~80% confluence in flask. Total cell numbers were counted using a coulter cell counter at the indicated times. For colony formation assay, 800 cells per well were seeded in a 6-well plate and cultured for 7–14 days in a 37°C incubator with medium changes every 3 days. The cells were fixed with 10% formalin and stained with 0.2% crystal violet (in 10% formalin) for 15 minutes. The cells were then washed followed by counting the number of cell colonies. The assays were performed in triplicate and all experiments were repeated more than three times.

### Assays for lactate production and glucose consumption

Cells recovered from either the Matrigel tube or cryogenic vial were cultured as described above. For lactate production and glucose consumption measurement, cells at 2 × 10^5^ per well were grown in 6-well plates with DMEM and 10% FBS. Cells were shifted into 2 ml fresh media per well for 24 hours before measuring glucose and lactate level in the medium by Lactate Colorimetric Assay Kit (Biovision) and Glucose (GO) Assay kit (Sigma). For cell in vivo lactate level, Cells were washed with PBS and homogenized in the assay buffer. Lactate level was then determined according to instructions of manufacture.

### MTT cell viability assay

The effect of chemotherapeutic agents on cell viability was measured by MTT assay. Briefly, 3,000 cells were seeded in 96-well plates and cultured for 24 hours before different concentrations of taxol or doxorubicin were added. Cell viability was examined at 48 hours after treatment. MTT assay was performed according to the manufacture’s instruction (Sigma).

### Statistical Analysis

The data are presented as mean values ± SD from five to eight independent experiments. Data analysis was performed using two-tailed Student’s t tests and *p* value are shown. *, *p <* 0.05; **, *p <* 0.01; N.S., not significant.

## Results

**Fig 2:**
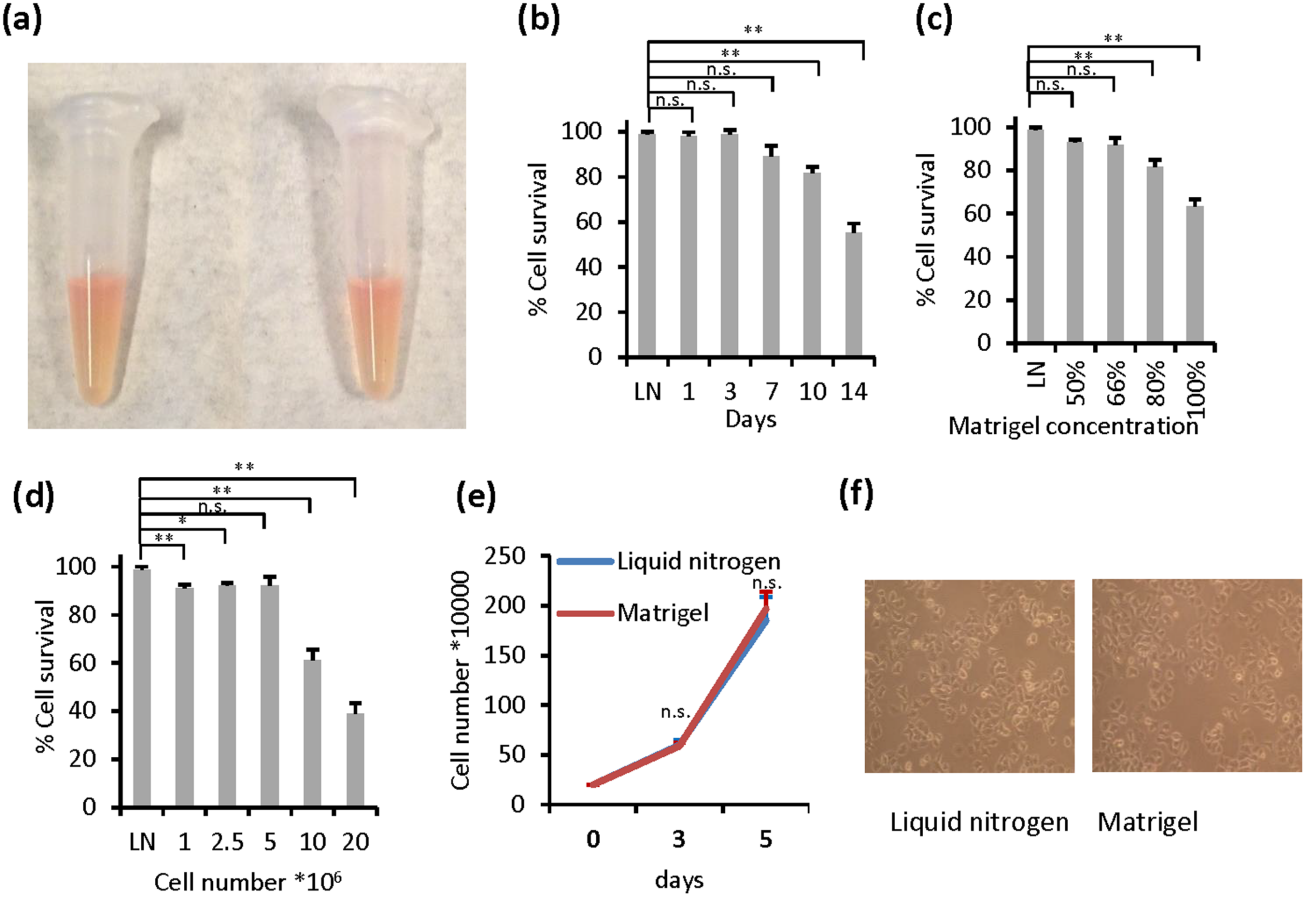
The survival rate of MCF-7 cells stored in the cell growth medium supplemented Matrigel. Cells were preserved in cryogenic vials in liquid nitrogen (LN) for 14 days, and were thawed and used as a control for determined cell viability by trypan blue exclusion assay. (a) Images of cells stored in medium supplemented Matrigel in Eppendorf tubes. (b) Cells were recovered from medium (34%) supplemented Matrigel (66%) as described in Materials and Methods, the cell survival rate was determined at the indicated time. (c) Cells were preserved in the mixture with different ratios between medium and Matrigel for 7 days, and were recovered for determining cell survival rate. (d) Different densities of cells were preserved in medium (34%) supplemented Matrigel (66%) for 7 days, and were recovered for determining cell survival rate. (e) and (f) Cells were preserved in medium (34%) supplemented Matrigel (66%) for 7 days, and were recovered for cell growth assay as described in Materials and Methods. Representative images showed cellular morphology. The data are presented as the mean ± SD *, *p <* 0.05; **, *p <* 0.01; n.s., not significant.

In an experiment involving establishment of xenograft tumors, we accidentally found that leftover cells from injection, in the form of 50% Matrigel mixture, were highly viable after 4 days at room temperature. This observation prompted us to postulate that Matrigel might be useful in transporting cells in semi-solid gel condition at ambient temperatures. To test the hypothesis, MCF-7 breast cancer cells were resuspended in the mixture of 66% Matrigel and 34% cell culture medium in 1.5 ml-Eppendorf microcentrifuge tubes, and the mixture was allowed to gel at 37°C for two hours (Fig 2a). The tubes were left stand at bench at room temperature for different days, cells in the gel were then resuspended in complete cell growth medium to recover the cells (for details, see Materials and Methods). Cell viability was then determined by trypan blue exclusion assay. Remarkably, cells mixed with Matrigel and stored at room temperature maintained excellent viability for 1 week, which was comparable to cells cryopreserved in liquid nitrogen (Fig 2b). To identify the optimal conditions that gave the highest viability, different numbers of cells were mixed in different concentrations of Matrigel. The results showed that cell densities ranging from 1×10^6^ to 5×10^6^ cells per ml and Matrigel:medium ratios from 3:1 to 2:1 were optimal for cell viability (Fig 2c and 2d). Cell densities higher than 1×10^7^ showed lower survival rate. Matrigel alone (*i*.*e*., undiluted, without medium) was not an optimal condition. Moreover, cells recovered from the Matrigel mixture displayed morphology and cell growth rates that were essentially identical to cells thawed from cryovials in liquid nitrogen (Fig 2e and 2f). These results demonstrate that the cell growth medium-supplemented Matrigel provides cells with a suitable survival environment for maintaining their viability at room temperature.

The fact that cells survived well at room temperature in an Eppendorf tube suggests that the condition identified could be a convenient way of transporting cells at ambient temperature. To demonstrate that the conditions we identified can be used for shipping cells, MCF-7 cells were resuspended in the medium-supplemented Matrigel, aliquoted into an Eppendorf tube and then sent via FedEx from our laboratory located at the UC Davis Medical Center, Sacramento, California to a laboratory at the MD Anderson Cancer Center, Houston, Texas (3 days transit), to a laboratory at the University of Minnesota, Minneapolis, Minnesota (4 days transit) and to a laboratory at the Guangzhou University of Chinese Medicine, Guangzhou, China (4 days transit) at ambient temperature. Upon arrival, the receiving laboratories recovered the cells by following the procedures described in the Materials and Methods and tested their viability. They found that over 90% of the cells were viable upon arrival and grew well after replating. Therefore, our Matrigel-based method of storage cell can be a convenient way of shipping cells at ambient temperature.

**Fig 3:**
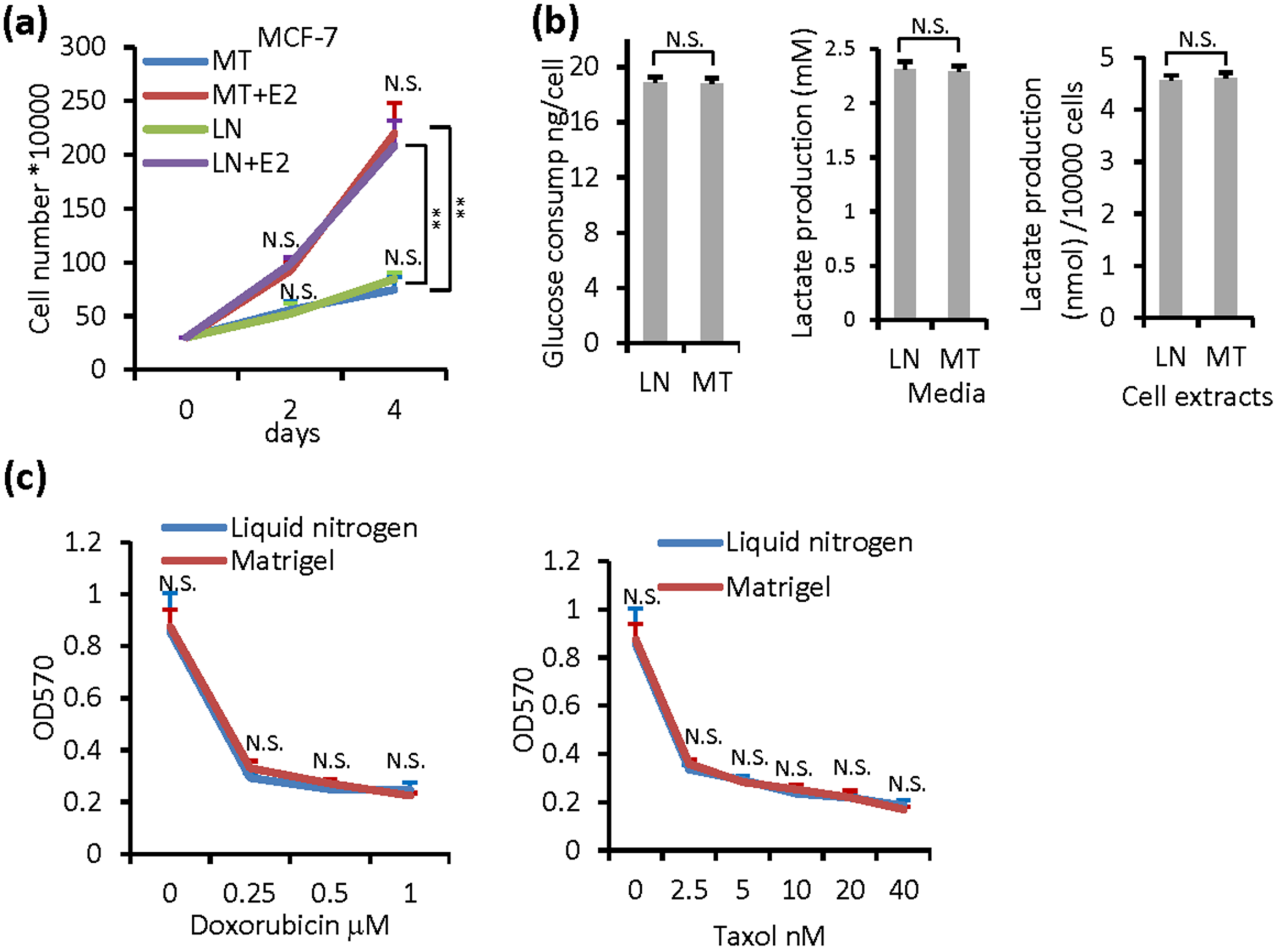
Comparison of properties of cells recovered from medium supplemented Matrigel (MT) and thawed from cryopreserved vials in liquid nitrogen (LN). MCF-7 cells were preserved in medium (34%) supplemented Matrigel (66%) for 7 days, and were recovered as described in Materials and Methods. Cryopeserved cells in liquid nitrogen as Fig 2 was used as a control. (a) Growth of MCF-7 cells treated with hormone (estradiol, *E2*) or vehicle was assayed. (b) Glucose consumption and lactate production were detected as described in material and methods. (c) Cell growth was detected by MTT assay after cells treated with chemotherapy drug doxorubicin and taxol for 48 hours. The data are presented as the mean ± SD, **, *p <* 0.01; N.S., not significant.

We next performed experiments to further characterize cells recovered from seven days of storage in the medium-supplemented Matrigel. MCF-7 cells are estrogen receptor-positive breast cancer cells and highly sensitive to estrogen in growth stimulation. Upon recovery, cells from the medium-supplemented Matrigel and from the cryovials showed essentially identical slow growth curves in the absence of estrogen and faster growth curves with estrogen (Fig 3a). Aberrant metabolism is a hallmark of cancer [10]. Tumor cells display a heightened glucose uptake, glycolysis, and lactate production. Cells recovered from the medium-supplemented Matrigel and from cryostorage showed the similar a glucose uptake and lactate production (Fig 3b). MCF-7 cells are also sensitive to growth inhibition by chemotherapeutic drugs taxol and doxorubicin [11,12]. Cells recovered from the medium-supplemented Matrigel and from the cryovials were growth-inhibited in an identical manner by doxorubicin and taxol (Fig 3c). These experiments illustrated that the method of Matrigel-based transporting of cells can preserve the cancer cell properties well.

**Fig 4:**
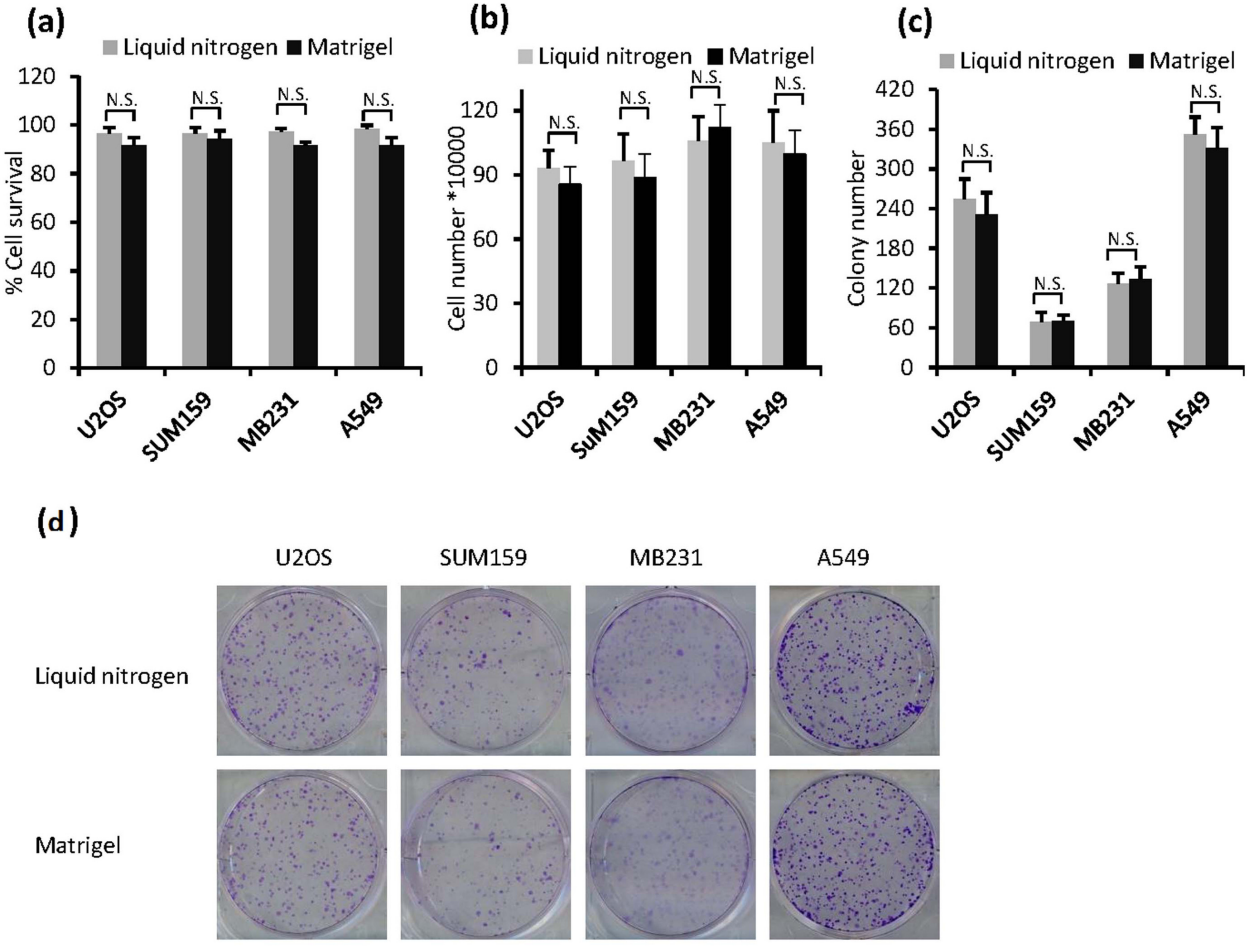
The survival rate of various types of cells recovered from medium supplemented Matrigel and thawed from cryopreserved vials in liquid nitrogen. Various types of cells at 5×10^6^ per ml were cryopreserved in cryogenic vial in liquid nitrogen or stored in medium (34%) supplemented Matrigel (66%) at room temperature for 7 days, cells were then recovered for assays. (a) Cell survival rates were detected by Trypan blue exclusion assay. (b) Cell growth assay were done as described in Fig 2(e). (c) and (d) Cell colony formation were determined as described in material and methods. The data are presented as the mean ± SD, *, *p <* 0.05; N.S., not significant.

Next, we examined whether this Matrigel-based method can be used for other types of cancer cells. Thus, A549 (lung cancer), MDA-MB231 and SUM159 (breast cancer), and U2OS (osteosarcoma) cells were stored in the medium-supplemented Matrigel at room temperature for 7 days and then assayed for their viability by trypan blue exclusion assay and colony formation assay. Remarkably, all of the cells showed high viability and cell morphology that were highly similar to those of cryopreserved cells (Fig 4, S1 and S2 Figs). These results indicate that the Matrigel-based method can preserve high levels of cell viability and is highly suitable for transportation of various types of cancer cells.

## Discussion

Recently, several studies intended to find suitable materials and methods for transporting cells without dry ice. In one method, cells attached to and growing in plates were covered with 1% agarose-medium mixture at 45°C, which can maintain viability up to 6 days for the cells tested [13]. One drawback is that many types of cells do not survival well due to the high temperature of the melted agarose (data not shown), furthermore, agarose gel often detached from plate during shipping. In another method, gelatin solutions were carefully prepared from animal skin and used to cover growing NIH3T3 cells in plates and found have an excellent activity to maintain the cell viability at either 10°C or 23°C. However, the medium-gel mixture melts at 37°C, which can limit its use for shipping at ambient temperature around 37°C. Also it is unclear if the method is effective in preserving viability for other cell types since only NIH3T3 cells were tested in the study [14]. Moreover, methods involving more elaborate procedures and materials were also reported for cryopreservation or storage of more specific cell types [15,16]. Their practical use and convenience is unclear. The Matrigel-based method we described here has been successfully used many times for shipping cells at ambient temperature by us and other laboratories using international and domestic service of FedEx. Therefore, our method can be practically applied. Moreover, it does not require special processing and packaging. Therefore, our method is highly convenient for shipping cells over long distances, including intercontinental shipping.

## Supporting Information

S1 Fig Representative images showed morphology of cells recovered from medium supplemented Matrigel and thawed from cryopreserved vials in liquid nitrogen. Various types of cells were stored in medium (34%) supplemented Matrigel (66%) at room temperature or cryogenic tubes in liquid nitrogen for 7 days, and were recovered. Representative images showed cellular morphology.
(PDF)

S2 Fig The cell growth rate of various types of cells recovered from medium supplemented Matrigel and thawed from cryopreserved vials in liquid nitrogen. Various types of cells were stored in medium (34%) supplemented Matrigel (66%) at room temperature or cryogenic tubes in liquid nitrogen for 7 days, and were recovered for determining cell growth.
(PDF)
