# Supplementary material for: Transporting Cells in Semi-Solid Gel Condition and at Ambient Temperature
Source: PLoS One. 2015 Jun 22;10(6):e0128229. doi: 10.1371/journal.pone.0128229 (PMC4476595; doi:10.1371/journal.pone.0128229)
Supplement: S1 Fig — Various types of cells were stored in medium (34%) supplemented Matrigel (66%) at room temperature or cryogenic tubes in liquid nitrogen for 7 days, and were recovered. Representative images showed cellular morphology. (PDF) [file pone.0128229.s001.pdf]

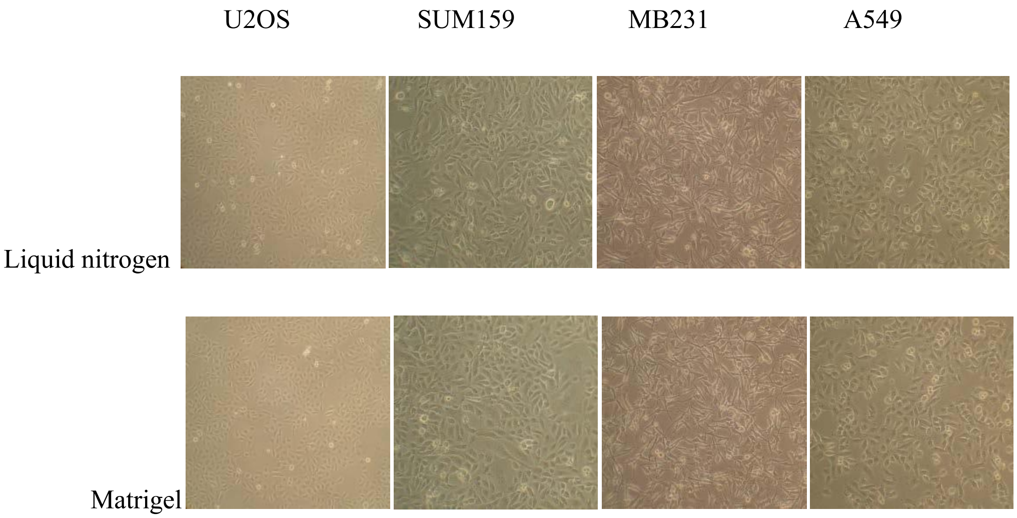

S1 Fig. Representative images showed morphology of cells recovered from medium supplemented Matrigel and thawed from cryopreserved vials in liquid nitrogen. Various types of cells were stored in medium (34%) supplemented Matrigel (66%) at room temperature or cryogenic tubes in liquid nitrogen for 7 days, and were recovered. Representative images showed cellular morphology.
