# Supplementary material for: Transporting Cells in Semi-Solid Gel Condition and at Ambient Temperature
Source: PLoS One. 2015 Jun 22;10(6):e0128229. doi: 10.1371/journal.pone.0128229 (PMC4476595; doi:10.1371/journal.pone.0128229)
Supplement: S2 Fig — Various types of cells were stored in medium (34%) supplemented Matrigel (66%) at room temperature or cryogenic tubes in liquid nitrogen for 7 days, and were recovered for determining cell growth. (PDF) [file pone.0128229.s002.pdf]

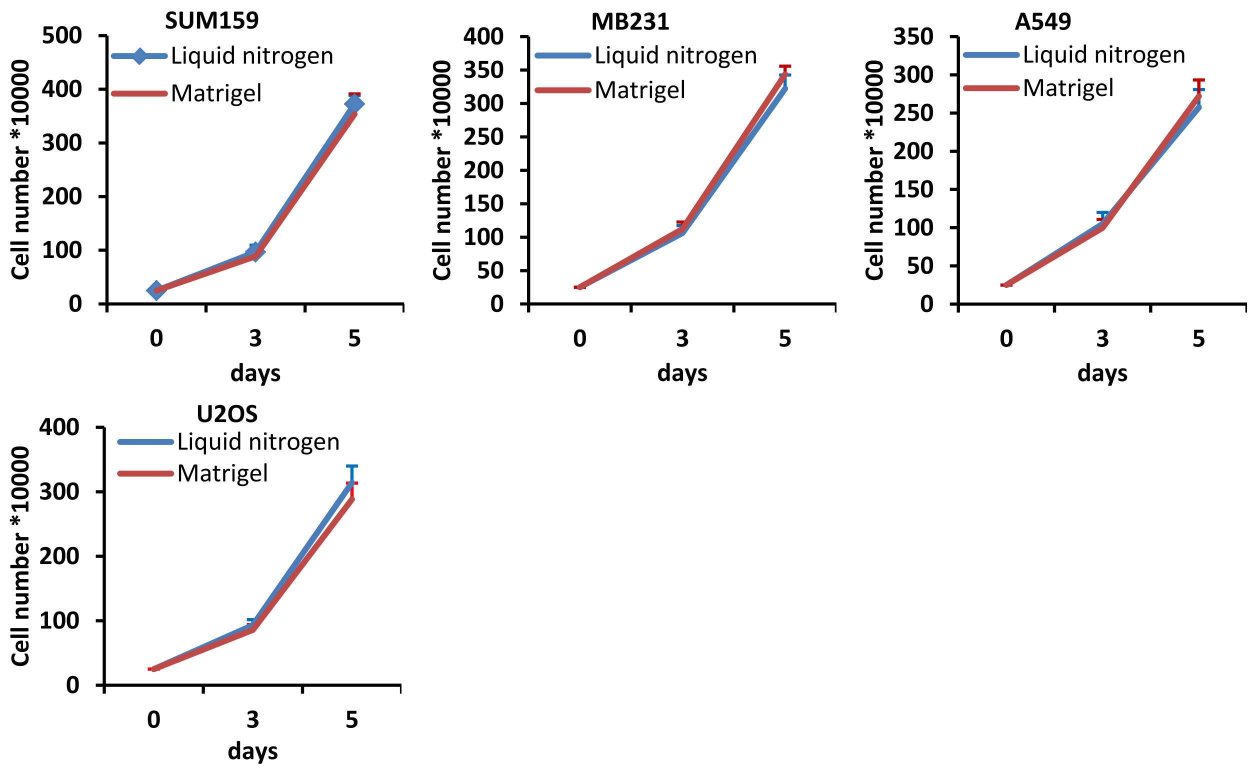

S2 Fig. The cell growth rate of various types of cells recovered from medium supplemented Matrigel and thawed from cryopreserved vials in liquid nitrogen. Various types of cells were stored in medium (34%) supplemented Matrigel (66%) at room temperature or cryogenic tubes in liquid nitrogen for 7 days, and were recovered for determining cell growth.
